# Health-Related Quality of Life in Older Adults: Its Association with Health Literacy, Self-Efficacy, Social Support, and Health-Promoting Behavior

**DOI:** 10.3390/healthcare8040407

**Published:** 2020-10-16

**Authors:** Myung Kyung Lee, Jihyun Oh

**Affiliations:** 1 College of Nursing, Research Institute of Nursing Science, Kyungpook National University, Daegu 41944, Korea; mlee@knu.ac.kr; 2 Department of Nursing, Daejeon University, Daejeon 34520, Korea

**Keywords:** health literacy, self-efficacy, social support, health-promoting behavior, quality of life

## Abstract

This cross-sectional study aimed to explore the relationships among sociodemographics, health literacy, self-efficacy, social support, health-promoting behavior, and health-related quality of life (HRQOL) in older adults. A total of 240 older adults aged >65 years were recruited from three community senior welfare centers in South Korea. Standardized self-administered questionnaires measuring sociodemographic characteristics, health literacy, social support, self-efficacy, health-promoting behavior, and health-related quality of life were distributed to older adults. Multiple regression analyses with stepwise selection was used to determine the factors affecting health-related quality of life. Factors affecting a higher physical component score of HRQOL were a higher comprehension level of and numeracy in health literacy, physical health-promoting behavior, perceived emotional-informational support, and a lesser number of comorbidities. Factors affecting a higher mental component score of HRQOL were a higher comprehension level of and numeracy in health literacy, self-efficacy, physical health-promoting behavior, perceived emotional-informational support, and a lesser number of comorbidities. To improve HRQOL among older adults, nursing interventions are required to measure health literacy, empower physical health-promoting behavior and self-efficacy, and enhance emotional-informational support from family or other resources.

## 1. Introduction

The elderly group is the fastest growing segment of the population, predicted to rise from 15.7% in 2020 to 25.0% in 2030 [1]. Cognitive and psychosocial factors have been shown to determine survival, use of medical services, health-promoting behavior, and health-related quality of life (HRQOL) in older individuals [2,3,4,5]. As the key cognitive and psychosocial factors, health literacy, self-efficacy, social support, and health-promoting behavior are considered in this older age group. 

Health literacy (HL), defined as the capacity to access, understand, evaluate, and apply basic health information to promote, maintain, and improve one’s health during the course of life [6], plays a significant role in managing multiple chronic diseases in older adults. In South Korea, over 90% of adults aged >70 years have low literacy skills with a low educational level, defined as having low HL [7,8]. Low HL is associated with negative health outcomes, including low HRQOL in older adults [4,9], poor use of healthcare in older adults [10], misunderstanding medical instructions, unhealthy behaviors in older adults [11], poor medication adherence in older adults with asthma [12], and poorer self-reported health in the elderly [9]. 

Self-efficacy is a useful indicator of general adaptational outcomes [13]. Psychological factors play a key role in the adjustment of older adults to chronic disease. Self-efficacy refers to an individual’s belief in his or her capacity to execute behaviors necessary to produce specific performance attainments [14]. A person with high self-efficacy can have a more active life course [15], and the active approach to life may have a positive effect on an individual’s QOL. Individuals with high, generalized self-efficacy are more likely to benefit from health education programs than those with low self-efficacy in healthy older people [16]. Previous studies found that general self-efficacy and specific self-efficacy for specific disease self-management (e.g., diabetes or stoma management) is correlated with a positive QOL in older adults or older patients [3,17,18] and improved compliance with self-care activities in older patients [19]. Moreover, social support is the perception and experience that one is valued, respected, cared for, and loved by others who are present in one’s life [20]. Social support has been recognized as one of the most important factors of QOL in older adults [21]. It has a significant effect on the QOL of the older adults [18,21] and chronically ill patients [2]. Thus, the provision of social support from family, healthcare providers, friends, and peer group has been suggested to be employed in the intervention programs for the older adults, chronically ill older patients, or vulnerable older adults as an important strategy in the intervention for improving QOL [22,23]. 

Moreover, health-promoting lifestyles are the primary adjustable strategy focusing on health promotion through lifestyle, which is vital to the quality of life. A health-promoting lifestyle includes activities to maintain or improve one’s health, including physical, social, and emotional well-being, and to affect healthy aging [24]. A healthy lifestyle leads to better health outcomes of the remaining years of the elderly individual [25]. Previous studies demonstrated that a healthy lifestyle not only reflects a subjective health status but also decreases the development of chronic disease and mitigates chronic medical conditions in older patients [2,26].

As mentioned above, the importance of HL, self-efficacy, social support, and health-promoting behavior for successful aging, which significantly affect older adults’ QOL, is well documented [2,3,4,5,22,27,28]. These are modifiable variables that can affect health status and QOL.

Some studies demonstrated that QOL is not significantly correlated with HL [29,30,31], self-efficacy [26], or health-promoting behavior [32,33] in older adults. In contrast, other studies showed that higher levels of HL, self-efficacy, social support, or health-promoting behavior in older adults were associated with higher QOL scores [2,3,4,5,20,24,25,26,34]. Previous studies indicated that results from the studies of the relationships of HL, self-efficacy, social support, and health-promoting behavior to HRQOL have substantially less consistency. Repeated studies on these factors affecting QOL are needed. Identifying the research gap in cognitive and psychosocial factors associated with HRQOL is essential for the development of health intervention in nursing practice for older adults.

According to the above-described background, the research question of this study was whether health literacy, self-efficacy, health-promoting behavior, and social support perceived by older adults were associated with their health-related quality of life, and to find answers to the research question, a cross-sectional design survey was conducted. Our hypothesis was that the higher health literacy, self-efficacy, health-promoting behavior, and social support perceived by older adults has a positive effect on their health-related quality of life. Therefore, this study aimed to determine the relationships among health literacy, self-efficacy, social support, health-promoting behavior, and HRQOL in older adults and to investigate their effects on HRQOL.

## 2. Materials and Methods

A cross-sectional survey was conducted at three community senior welfare centers in the central district of South Korea from 27 November 2016 to 5 January 2017. Participants were recruited through convenience sampling when they visited community centers. The inclusion criteria were as follows: (1) age of at least 65 years; (2) no cognitive impairment detected during Mini-Cog instrument screening [35]; and (3) ability to communicate and complete a self-administered questionnaire. Questionnaires were directly distributed only to older adults who had provided written consent after being informed about the purpose of the study. The survey was distributed to 249 older adults, of which 240 questionnaires were returned (response rate: 96.3%). In principle, the participants answered the questionnaire by themselves, and if they asked for help or a question while answering the questionnaire, the research staff provided support. In the case of the subjects’ vision problems, the researcher staff read the questions of the study and marked the subjects’ answers.

### 2.1. Measurement

#### 2.1.1. Health Literacy

The short form of the Korean Health Literacy Scale (S-KHLS), developed by Lee and Kang [36], was used to measure HL. The S-KHLS consists of 12 items, with five questions on health-related terms and seven questions on comprehension and numeracy. The Cronbach’s α for this scale in this study was 0.80.

#### 2.1.2. Social Support

Social support was measured using the 19-item Medical Outcome Study Social Support Survey (MOS-SSS), developed by Sherbourne and Stewart [37]. The items measure the four subscales of social support using a 5-point Likert response scale, ranging from 1 (none of the time) to 5 (all of the time). The MOS-SSS is designed to assess four different dimensions of social support: emotional-informational, tangible support, affectionate support, and positive social interaction. The total score ranges from 0 to 100, in which higher scores indicate higher social support and lower risk of isolation. The Cronbach’s α for this scale in this study was 0.96.

#### 2.1.3. Self-Efficacy

The self-efficacy scale was developed by Schwarzer and Jerusalem [15]. This scale consists of 10 items using a 4-point Likert scale, ranging from 1 (not at all true) to 4 (completely true). The total score ranges from 10 to 40, with a higher score indicating higher self-efficacy. The Cronbach’s α in the current study was 0.89, suggesting acceptable internal consistency.

#### 2.1.4. Health-Promoting Behavior

The Health Promotion Lifestyle Profile II, developed by Walker et al. [38] and modified by Lee [21], was used to measure the health-promoting behavior. The scale consisted of 13 items that included three dimensions of health-promoting behavior: physical area (five items), social area (five items), and emotional area (three items). The scale was scored on a 5-point Likert scale, with responses ranging from 1 (strongly disagree) to 5 (strongly agree). The total score ranges from 13 to 65, with higher scores representing a higher level of health-promoting behavior. The Cronbach’s α for this scale in this study was 0.85.

#### 2.1.5. Health-Related Quality of Life

This scale was assessed using the Medical Outcomes Study Short-Form Health Survey-12 (SF-12) [39]. The SF-12 is an abbreviated version of the original SF-36. This instrument contains 12 items, which measure two components of QOL: Physical Component Summary (PCS-12) and Mental Component Summary (MCS-12). The scores range from 0 to 100, and a higher score indicates better HRQOL. The Cronbach’s α for this scale in this study was 0.86.

### 2.2. Ethical Considerations

The study was approved by the University Institutional Review Board (number: 1040647-201601-HR003-03). All participants were informed about the aims and plan of the study, and written consent was obtained from all study participants prior to their participation. Moreover, this study complied with the ethical guidelines delineated in the Declaration of Helsinki.

### 2.3. Statistical Analyses

Sociodemographic characteristics, HL, perceived social support, self-efficacy, health-promoting behavior, and HRQOL were analyzed using descriptive statistics. For the univariate analyses, independent *t*-test, one-way analysis of variance (ANOVA), and Scheffé’s post hoc test were performed to compare the HRQOL according to sociodemographic parameters. Correlations among HL, social support, self-efficacy, and health-promoting behavior were identified using Pearson’s correlation coefficient.

We performed multiple regression analyses with stepwise selection to determine the factors affecting HRQOL, in which we set a regression model with the variables that were significantly associated with HRQOL in the univariate analyses (i.e., independent *t*-test, ANOVA, Pearson’s correlation). All statistical analyses were two sided; *p*-values < 0.05 were considered statistically significant. All statistical analyses were performed using IBM SPSS Statistics software Version 22.0 (IBM Corp., Armonk, NY, USA).

## 3. Results

### 3.1. Participants’ General Characteristics and Differences

Of 249 individuals, 240 fully participated in the study (response rate, 96.3%) (Table 1). The mean age of the participants was 75.6 years (standard deviation (SD) = 6.3), ranging from 65 to 95 years. More than half of the participants were male (57.9%), 65.0% were married, and the proportion of those who did not receive education or elementary school was 43.3%. A majority of participants (69.6%) were affiliated with a religious group. More than half of the participants (58.8%) reported their economic status as above mid-level, and 46.7% had at least one comorbidity. Regarding the PCS scores according to participants’ general characteristics, there was a significant difference in age group (t = 2.652, *p* = 0.008), marital status (F = 3.92, *p* = 0.021), economic status (t = 2.60, *p* = 0.010), and number of comorbidities (F = 13.82, *p* < 0.001). Regarding the MCS scores according to participants’ general characteristics, there was a significant difference in economic status (t = 2.76, *p* = 0.006) and number of comorbidities (F = 6.22, *p* = 0.002). The results of the post hoc tests with ANOVA showed that older adults who had more than one comorbidity showed significantly lower PCS scores than those without comorbidities. Moreover, older adults who had more than one comorbidity showed significantly lower MCS scores than those without comorbidities.

### 3.2. Scores for HL, Social Support, Self-Efficacy, Health-Promoting Behavior, and HRQOL

As shown in Table 2, the mean comprehension and numeracy scores for HL was 5.0 out of a maximum possible score of 7, and the mean health-related term score for HL was 4.3 out of a maximum possible score of 5. Of the social support subscales, the scores for tangible support, affectionate support, positive social interaction, and emotional-informational support were 14.8 (SD = 4.7), 11.5 (SD = 3.4), 14.9 (SD = 4.5), and 30.0 (SD = 5.8), respectively. The mean score for self-efficacy was 30.8 (SD = 5.8). Of the health-promoting behavior subscales, the physical area showed the highest score (M = 21.4, SD = 3.6), followed by the social area (M = 18.0, SD = 4.5), and the emotional area showed the lowest score (M = 11.7, SD = 2.7). The mean HRQOL score for physical health was 63.6 (SD = 7.8), whereas that for mental health was 67.8 (SD = 10.8).

### 3.3. Correlations between HRQOL and HL, Social Support, and Health-Promoting Behavior

The physical component of HRQOL was significantly positively correlated with comprehension and numeracy subscale of HL (*r* = 0.16, *p* = 0.011), all subscales of social support (tangible support, *r* = 0.27, *p* < 0.001; affectionate support, *r* = 0.20, *p* = 0.002; positive social interaction, *r* = 0.25, *p* < 0.001; emotional-informational support, *r* = 0.30, *p* < 0.001), self-efficacy (*r* = 0.23, *p* < 0.001), and all subscales of health-promoting behavior (physical area, *r* = 0.28, *p* < 0.001; social area, *r* = 0.21, *p* < 0.001; emotional area, *r* = 0.18, *p* = 0.004).

The mental component of HRQOL was significantly positively correlated with all subscales of HL (health-related terms, *r* = 0.13, *p* = 0.047; comprehension and numeracy, *r* = 0.20, *p* = 0.002), all subscales of social support (tangible support, *r* = 0.24, *p* < 0.001; affectionate support, *r* = 0.31, *p* < 0.001; positive social interaction, *r* = 0.29, *p* < 0.001; emotional-informational support, *r* = 0.36, *p* < 0.001), self-efficacy (*r* = 0.30, *p* < 0.001), and all subscales of health-promoting behavior (physical area, *r* = 0.23, *p* < 0.001; social area, *r* = 0.17, *p* < 0.001; emotional area, *r* = 0.18, *p* = 0.006) (Table 3).

### 3.4. Factors Affecting HRQOL

Factors affecting a higher physical component score of HRQOL were higher comprehension and numeracy of HL (β = 0.40, *p* = 0.022), physical health-promoting behavior (β = 0.30, *p* < 0.001), perceived emotional-informational support (β = 0.11, *p* = 0.001), and a lesser number of comorbidities (β = −1.66, *p* < 0.001). These factors explained 21% of the total variance of the physical component of HRQOL.

Factors affecting a higher mental component score of HRQOL were higher comprehension of and numeracy in HL (β = 0.59, *p* = 0.001), self-efficacy (β = 0.16, *p* = 0.002), physical area (β = 0.16, *p* = 0.040), perceived emotional-informational support (β = 0.13, *p* = 0.001), and less number of comorbidities (β = −0.78, *p* = 0.004). These factors explained 21% of the total variance of mental component of HRQOL (Table 4). The results showed that higher health literacy, self-efficacy, health-promoting behavior, and social support had a positive influence on older adults’ HRQOL, and our hypothesis was accepted.

## 4. Discussion

The primary aim of the current study was to examine the factors affecting the HRQOL of older adults. Five variables were found to be significantly associated with HRQOL. Our results suggest that reading comprehension of and numeracy in HL, health-promoting behavior in the physical area, and emotional-informational support were significantly associated with the physical and mental components of HRQOL. Self-efficacy was also associated with the mental component of HRQOL. Furthermore, the number of comorbidities was associated with the mental and physical components of HRQOL. Our findings confirmed the results of previous studies.

In this study, the mean scores for MCS and PCS of HRQOL were 63.6 (7.8) and 67.8 (10.8) out of 100 points, respectively. The mean scores of HRQOL within our sample were considerably higher than the mean scores of MCS and PCS of elderly patients hospitalized for cardiovascular disease [40] and long-term dialysis patients [41]. This finding suggested that with the progression of the chronic diseases, health intervention should include different chronic disease programs to manage chronic conditions and strengthen health status.

The present findings are consistent with those of previous studies, suggesting that HL had a positive influence on HRQOL [30,42]. However, inadequate reading comprehension and numeracy skills can inhibit a patient’s ability to attend appointment schedules with their clinicians and follow the prescription instructions correctly [43]. Misunderstanding medication labels and health messages is related to a higher adverse drug reaction and has been repeatedly found to be negatively associated with health outcomes [44,45]. With age, older individuals may have difficulty in performing complex cognitive tasks, and this could have resulted in poor HL, which may be related to poor health outcomes [46]. The results of this study support the findings of previous studies [47,48] that good HL results in better HRQOL.

The expected association between self-efficacy and the mental component of HRQOL was confirmed. This finding is in accordance with those of former studies, which indicated that greater self-efficacy was more strongly related to positive HRQOL of older adults [17,18,49,50]. Specific self-efficacy and general self-efficacy predicted global HRQOL. Specific self-efficacy on diabetes management (a patient’s ability and confidence to effectively manage diabetes [51]), pain management [50,52,53], or stoma management [17] may be useful in improving HRQOL in this population. Moreover, general self-efficacy is important and independently correlated with HRQOL in older adults [3,18,49]. Our study specifically showed positive association between general self-efficacy and the mental health component of HRQOL in older adults. Self-efficacy, which refers to a patient’s confidence in their ability to successfully achieve desired behavioral outcomes, is consistent with taking control and self-advocacy [14]. Bandura (1999) indicated that self-efficacy determines whether individuals think of their emotional well-being in self-aiding or self-debilitating ways [14]. One previous study suggested that individuals with high self-efficacy have less health-related stress [54]. Another study indicated that higher self-efficacy is associated with positive emotions and optimism [55], which may also partially explain the positive impact of self-efficacy on the mental component of HRQOL. Older individuals with higher self-efficacy can gain an increased sense of confidence in their ability to control and manage symptoms associated with chronic disease, long-term adherence in managing their disease [56], and better coping skills with a disease [57], which significantly enhance HRQOL. This study suggested that incorporating the concept of self-efficacy when instructing older adults about healthcare and management could help improve their chances of attaining a higher HRQOL perception. Consequently, older adults’ beliefs regarding self-efficacy can affect their mental QOL. Hence, older individuals who aim to take responsibility for their self-care and believe that they have the skills in the desired care behavior have better mental QOL.

Similar to those of previous studies [2,28,58], our findings demonstrate a positive correlation between health-promoting behaviors and HRQOL in older adults. Among all health-promoting behaviors, the physical area was the only predictive factor for HRQOL. Age-related physical changes affect physical functions in older adults. With advancing age, health-promoting behaviors reduce the risk of adverse health outcomes. Specifically, we found that engaging in more physical health-promoting behaviors (i.e., regular and adequate levels of physical activity, adequate sleep, rest and relaxation, a balanced and healthy diet, and regular walking) have a greater impact on HRQOL.

Our findings support a positive relationship between emotional-informational support and HRQOL as described in former studies, indicating that social support is more strongly related to a higher HRQOL in older adults [18,21,22,28,34], older individuals with stoma [17], and older individuals with chronic disease [2,27,35,38,59]. The use of peer support programs (volunteer visits with clients) enforcing emotional-informational support significantly increased the levels of well-being among highly vulnerable, low-income older adults [23]. In older adults without impaired mobility who are not institutionalized and who live in a community, the main sources of perceived social support were family members and significant others [22,59,60,61] and friendship networks [62]. Strong family support was associated with better mental and physical HRQOL in older patients with multiple chronic conditions [59], and a strong friendship network was positively associated with physical HRQOL [59,63]. The protective and nursing role of the family toward their older members in need is typical of collectivist cultures, such as in South Korea, in which individuals maintain strong relationships with their families for life [64]. Thus, emotional-informational support from family members contributes indirectly to improved chronic disease-related outcomes and reduced psychological distress [65]. Moreover, the emotional-informational support from friends or other resources may reduce the physical impact of disease and lead to positive effects on the physical and mental components of HRQOL.

Older individuals with more comorbidities presented worse mental and physical HRQOL in our study, which is consistent with the results of previous studies [59,66,67]. Older adults with comorbidities are more prone to developing physical and psychological limitations. These results highlight the importance of assessing chronic diseases when caring for older adults.

### 4.1. Implication for Nursing Practice

These findings indicated that HL and physical health-promoting behaviors are significant factors in improving the physical and mental components of HRQOL in older adults. The present study suggests that self-efficacy may be a crucial consideration when managing chronic disease and achieving a healthy lifestyle. Considering these results, to enhance HRQOL, nurses should take on a significant role in measuring HL and empowering physical health behaviors in older adults who have at least one chronic health condition by applying for health-promoting programs. 

In clinical practice, nurses must plan and execute age-appropriate teaching strategies for the elderly by using familiar examples to promote health literacy. Healthcare providers, including nurses or nurse managers, must provide considerable effort to provide the elderly with health education and health information to improve their quality of living. 

### 4.2. Limitations

This study has several limitations. First, the sample of the study was a convenience sample of community-dwelling older adults. In addition, recruiting from only one rural area in South Korea may limit the generalizability of the results to other countries. Second, HL, perceived social support, self-efficacy, health-promoting behavior, and HRQOL were based on self-reports, and the present study is a cross-sectional study. Therefore, cause-and-effect interpretations on variables cannot be established.

## 5. Conclusions

The findings demonstrate that HL, self-efficacy, social support, and health-promoting behavior are important for an elderly individual’s QOL. Moreover, 21–22% of the variance in HRQOL outcomes were validated by the results obtained. However, there is still a notable proportion of unexplained variance. Additional research should be conducted to assess other predictors of HRQOL in older adults.

## Figures and Tables

**Table 1:** Descriptive statistics of participants’ general characteristics and differences (*N* = 240).

Characteristics	Mean	Range	*n* (%)	PCS-12		MCS-12	
				Mean	t or F (*p*) Scheffé	Mean	t or F (*p*) Scheffé
Age (year)	75.6 (6.3)	65–95					
≥65, <75			108 (45.0)	64.4 (14.9)		78.4 (15.9)	
≥75, <95			132 (55.0)	59.1 (15.9)	2.652 (0.008)	74.9 (15.2)	1.731 (0.085)
Sex							
Male			139 (57.9)	60.3 (15.9)		76.7 (75.1)	
Female			101 (42.1)	63.1 (15.2)	−1.34 (0.182)	76.2 (16.4)	0.23 (0.822)
Education							
No education or elementary school			104 (43.3)	59.3 (15.1)		75.2 (15.7)	
Middle school			51 (21.3)	62.4 (17.5)		76.9 (16.8)	
≥High school			85 (35.4)	63.6 (15.0)	1.84 (0.161)	77.8 (14.9)	0.64 (0.530)
Marital status							
Single			17 (7.1)	55.3 (20.9)		70.2 (22.4)	
Married			156 (65.0)	63.5 (14.5)		77.3 (15.1)	
Divorced/Widowed			67 (27.9)	58.5 (16.1)	3.92 (0.021)	76.2 (14.7)	1.60 (0.203)
Religion							
Yes			167 (69.6)	60.8 (15.9)		75.7 (15.9)	
No			73 (30.5)	63.0 (15.1)	−1.00 (0.319)	78.3 (15.0)	−1.18 (0.241)
Economic status							
≥Middle			141 (58.8)	63.7 (14.7)		78.8 (15.2)	
Low			99 (41.3)	58.6 (16.6)	2.60 (0.010)	73.2 (15.7)	2.76 (0.006)
Number of comorbidities							
0			75 (31.3)	68.6 (14.6)		81.6 (14.7)	
1			112 (46.7)	59.6 (14.9)		74.5 (15.1)	
≥2			53 (22.1)	55.4 (15.2)	13.82 (<0.001) a > b, c	73.4 (16.5)	6.22 (0.002) a > b, c

SD, standard deviation; SF-12, Short-Form Health Survey; PCS, Physical Component Summary; MCS, Mental Component Summary; a, No comorbidity; b, 1 comorbidity; c, ≥2 comorbidities.

**Table 2:** Scores for health literacy, social support, self-efficacy, health-promoting behavior, and health-related quality of life (HRQOL) (*N* = 240).

Variables	Range	Mean [SD]
Health literacy		
Health-related terms	0–5	4.3 (1.0)
Comprehension and numeracy	0–7	5.0 (1.6)
Social support		
Tangible	4–20	14.8 (4.7)
Affectionate	3–15	11.5 (3.4)
Positive social interaction	4–20	14.9 (4.5)
Emotional-informational	8–40	30.0 (8.2)
Self-efficacy	10–40	30.8 (5.8)
Health-promoting behavior		
Physical area	5–25	21.4 (3.6)
Social area	5–25	18.0 (4.5)
Emotional area	3–15	11.7 (2.7)
HRQOL (SF-12)		
Physical health (PCS-12)	36.7–90.0	63.6 (7.8)
Mental health (MCS-12)	37.5–97.5	67.8 (10.8)

SD, standard deviation; HRQOL, health-related quality of life; SF-12, Short-Form Health Survey.

**Table 3:** Correlations among variables (*N* = 240).

Variables	r (*p*)											
	Health Literacy		Social Support				Self-Efficacy	HPB			SF-12	
	1	2	3	4	5	6	7	8	9	10	11	12
1. Health-related terms	―											
2. Comprehension and numeracy	0.30 (<0.001)	―										
3. Tangible	0.06 (0.380)	0.05 (0.479)	―									
4. Affectionate	0.04 (0.563)	0.10 (0.131)	0.71 (<0.001)	―								
5. Positive social interaction	0.05 (0.427)	0.09 (0.151)	0.76 (<0.001)	0.85 (<0.001)	―							
6. Emotional-informational	0.08 (0.200)	0.14 (0.026)	0.74 (<0.001)	0.85 (<0.001)	0.83 (<0.001)	―						
7. Self-efficacy	0.03 (0.698)	−0.13 (0.040)	0.17 (0.008)	0.27 (<0.001)	0.21 (<0.001)	0.32 (<0.001)	―					
8. Physical area	0.05 (0.428)	−0.003 (0.967)	0.06 (0.337)	0.07 (0.316)	0.07 (0.316)	0.15 (0.023)	0.34 (<0.001)	―				
9. Social area	0.05 (0.447)	−0.06 (0.375)	0.19 (0.003)	0.25 (<0.001)	0.29 (<0.001)	0.29 (<0.001)	0.30 (<0.001)	0.44 (<0.001)	―			
10. Emotional area	0.07 (0.285)	−0.07 (0.273)	0.13 (0.052)	0.14 (0.027)	0.09 (0.151)	0.18 (0.005)	0.19 (0.004)	0.39 (<0.001)	0.28 (<0.001)	―		
11. PCS	0.05 (0.428)	0.16 (0.011)	0.27 (<0.001)	0.20 (0.002)	0.25 (<0.001)	0.30 (<0.001)	0.23 (<0.001)	0.28 (<0.001)	0.21 (<0.001)	0.18 (0.004)	―	
12. MCS	0.13 (0.047)	0.20 (0.002)	0.24 (<0.001)	0.31 (<0.001)	0.29 (<0.001)	0.36 (<0.001)	0.30 (<0.001)	0.23 (<0.001)	0.17 (<0.001)	0.18 (0.006)	0.64 (<0.001)	―

HL, health literacy; HPB, health-promoting behavior; HRQOL, health-related quality of life; SF-12, Short-Form Health Survey; 1, health-related terms; 2, comprehension and numeracy; 3, tangible; 4, affectionate; 5, positive social interaction; 6, emotional-informational; 7, self-efficacy; 8, physical area; 9, social area; 10,emotional area; 11, PCS, Physical Component Summary; 12, MCS, Mental Component Summary.

**Table 4:** Results of the stepwise multiple regression analysis for HRQOL (*N* = 240).

Variable	PCS			MCS		
	β (SE)	*t*	*p*	β (SE)	*t*	*p*
Constant	8.27 (2.03)	4.08	<0.001	8.49 (2.24)	4.08	<0.001
Reading comprehension and numeracy	0.40 (0.17)	2.31	0.022	0.59 (0.18)	3.34	0.001
Self-efficacy	―	―	―	0.16 (0.05)	3.09	0.002
Physical area	0.30 (0.08)	3.92	<0.001	0.16 (0.08)	2.07	0.040
Emotional-informational	0.11 (0.03)	3.28	0.001	0.13 (0.03)	3.48	0.001
Number of comorbidities	−1.66 (0.38)	−4.37	<0.001	−0.78 (0.38)	−2.06	0.004
Adjusted R^2^	0.212			0.216		

HRQOL, health-related quality of life; PCS, Physical Component Summary; MCS, Mental Component Summary; SE, standard error.
